# Magneto-Transport Properties of Co–Cu Thin Films Obtained by Co-Sputtering and Sputter Gas Aggregation

**DOI:** 10.3390/nano11010134

**Published:** 2021-01-08

**Authors:** Ricardo López Antón, Juan Pedro Andrés, Mihail Ipatov, Juan Antonio González, Julián González, Valentina Zhukova, Arcady Zhukov

**Affiliations:** 1 Departament of Applied Physics, Instituto Regional de Investigación Científica Aplicada (IRICA), University of Castilla-La Mancha, 13071 Ciudad Real, Spain; juanpedro.andres@uclm.es (J.P.A.); j.a.gonzalez@uclm.es (J.A.G.); 2 Departament of Advanced Polymers and Materials: Physics, Chemistry and Technology, Faculty of Chemistry, University of the Basque Country, 20018 San Sebastián, Spain; mihail.ipatov@ehu.eus (M.I.); julianmaria.gonzalez@ehu.eus (J.G.); valentina.zhukova@ehu.es (V.Z.); arkadi.joukov@ehu.es (A.Z.); 3 Department of Applied Physics I, EIG, University of the Basque Country, 20018 San Sebastián, Spain; 4 IKERBASQUE, Basque Foundation for Science, 48011 Bilbao, Spain

**Keywords:** Kondo effect, giant magnetoresistance, annealing, thin films, sputtering, sputter gas aggregation

## Abstract

Cu_100−x_Co_x_ thin films have been obtained by sputtering (x = 3, 9) and sputter gas aggregation (x = 2.5, 7.5) and subsequent annealing at 400 °C for 1 h. We have studied their structural, magnetic, and magnetotransport properties, both for the as-deposited and annealed samples, confirming the important role of the fabrication method in the properties. The magnetic measurements and the fitting of the hysteresis loops evidence that as-deposited samples consist of superparamagnetic (SPM) and/or ferromagnetic clusters, but in the samples obtained by gas aggregation the clusters are greater (with ferromagnetic behavior at room temperature) whereas in the samples obtained by sputtering, the clusters are smaller and there are also diluted Co atoms in the Cu matrix. The annealing affects negligibly the samples obtained by gas aggregation, but the ones obtained by sputtering are more affected, appearing greater clusters. This behavior is also reflected in the magnetoresistance (MR) measurements of the samples, with different shapes of the MR curves depending on the preparation method: more lineal in the whole range for sputtering, saturation at low fields (about 10 kOe) for gas aggregation. Finally, a Kondo-like minimum in the resistance versus temperature is found in the samples obtained by sputtering, affected by the magnetic field and the annealing. The observed Kondo-like behavior and the influence of annealing on a Kondo-like minimum in sputtered thin films have been attributed to the presence of diluted Co atoms in the Cu matrix and the Co precipitations from the Co–Cu solid solution upon annealing respectively.

## 1. Introduction

Since the discovery of giant magnetoresistance (GMR) in 1988 by Fert and Grünberg in magnetic multilayers [1,2], many materials with GMR have been developed. Nowadays, devices based on the GMR effect have been developed, which operate using two possible spin states (spin up and spin down) of electrons. The origin of the GMR effect is associated to spin-dependent electron scattering when travelling through a nanoscaled non-magnetic metallic spacer between two magnetic regions [1,2,3]. In particular, granular Co–Cu alloys are among them [3,4]. Co is practically immiscible in Cu at room temperature, but several preparation methods (as, e.g., sputtering, melt-quenching, electrodeposition, laser ablation, etc. [5,6,7,8,9]) allow to obtain a metastable solid solution of a small quantity of Co in Cu. In this situation, the (energetically more favorable) segregation of small precipitates of Co takes place (after annealing or, sometimes, even at preparation), giving rise to the so-called granular alloys. In those granular alloys, GMR appears because of spin-dependent scattering of the conducting electrons by the magnetic Co clusters [4,10].

Sputtering has been one of the most popular techniques used to obtain granular alloys presenting GMR (in fact, the pioneer works of Berkowitz [3] and Xiao [4] used this technique). On the other hand, combining sputter gas aggregation (allowing to form clusters of a controlled size) with sputtering (to deposit the matrix) has been used for obtaining granular alloys [11,12,13,14] although, in spite of allowing to control the size of the clusters, has been used scarcely for GMR studies [11,12,13]. Finally, melt-quenching has been broadly employed to obtain granular alloys, in the form of ribbons [5,15] and, more recently, in the form of microwires [16,17,18]. Noteworthily, in these microwires, in addition of the GMR effect, a minimum in the evolution of the resistance with the temperature has been found [16,17,18,19,20]. The typical explanation for such a resistance minimum is the Kondo effect related to magnetic impurities (in very low concentrations, below 0.1%) in metals [21]. The concentration of Co in the Cu matrix in those samples is quite higher, 5% or more, than those typical values but, nevertheless, the behavior of the minimum is quite close to the one observed in Kondo effect. Hence, the authors call this minimum a Kondo-like one.

The aforementioned Kondo-like behavior in Co–Cu granular alloys has been only observed in magnetic microwires prepared by the Taylor–Ulitovsky method involving rapid melt quenching [16,17,18,19,20] and recently also in Co/Cu superlattices with ultrathin Co layers (with Co thickness below 0.1 nm) [22]. It is also noteworthy that a similar behavior was also found in Fe/Cr multilayers (with very thin Fe layers) [23].

It is worth mentioning that, if the size of magnetic precipitates is small and even if they can be considered as magnetic impurities, the scattering of conduction electrons in a metal due to magnetic impurities give rise to a Kondo effect [21,24]. The Kondo effect, associated with the resonant scattering of conducting electrons by quantum local centers, is one of the key correlation effects in solid state physics [25]. The development of nanoscale electronic devices requires understanding and controlling of the behavior of electrons, in particular, correlation effects at the atomic scale. Therefore, in this work we have obtained Co–Cu granular alloys in the form of thin films by magnetron sputtering and gas aggregation, and we have studied the influence of the preparation method and annealing process on their magnetic and magnetotransport properties.

## 2. Experimental

**Figure 1:**
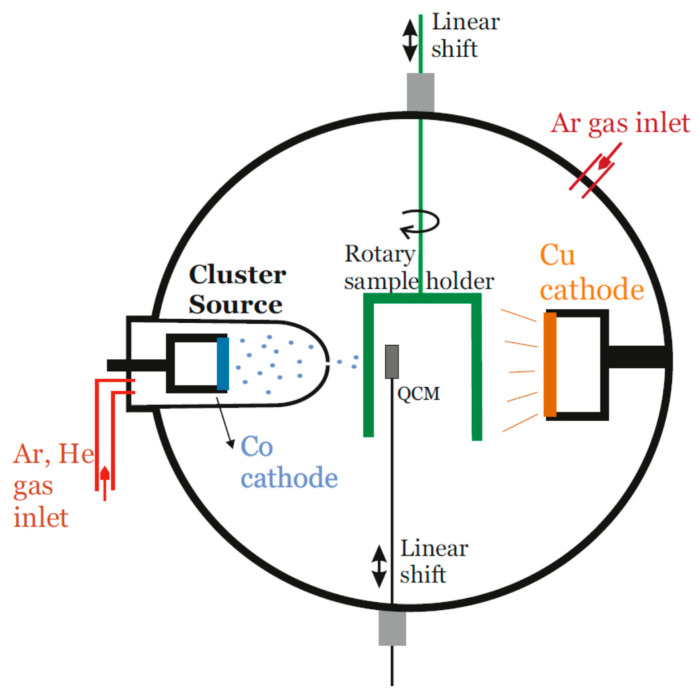
Schematical representation of the gas aggregation deposition system.

Cu_100−x_Co_x_ (x = 3, 9) thin films have been obtained by magnetron sputtering at room temperature using two different approaches. In the first one, conventional co-sputtering was used from a mosaic-like Co–Cu target with the adequate ratio of Co pieces on a Cu disc. In the second, Cu_100−x_Co_x_ (x = 2.5, 7.5) thin films were obtained by a combination of sputter gas aggregation of Co NPs in a separate chamber (i.e., a cluster source), and conventional magnetron sputtering of the Cu matrix in a second (deposition) chamber. Pure Co nanoparticles (NPs) were therefore pre-formed in the cluster source (with an Ar pressure of about 0.1 mbar) and then injected by differential pressure into the deposition chamber (with an Ar pressure 2 orders of magnitude lower) [26]. A cylindrical sample holder is continuously rotating at 14 rpm during deposition, allowing both components to be deposited quasi-simultaneously and forming the granular film of Co NPs embedded in a Cu matrix [14]. A schematic representation of the gas aggregation system is shown in Figure 1. The power of the cluster source is kept constant (50 W) whereas the power of the Cu target is changed, varying accordingly the ratio Co–Cu. The rate of deposition for Co NPs is evaluated by a quartz crystal monitor (QCM). The substrates used for the deposition are n-doped Si wafers, with (001) orientation and resistivity of 4–7 Ω·cm. The natural oxide layer on the surface was not removed.

The size of the Co NPs was checked by transmission electron microscopy (TEM), using JEOL JEM 2011 electron microscope (Tokyo, Japan) operating at 200 kV, performed on nanoparticles deposited on a TEM grid (figure not shown). The mean size of those NPs was about 10 nm. The composition of all the samples was obtained by EDX measurements in a scanning electron microscope (Zeiss GeminiSEM 500, Oberkrochen, Germany).

The samples were annealed in a high vacuum (about 10–5 mbar) for one hour at 400 °C (673 K). This temperature is enough to start the Co segregation and was chosen in order to better compare with the results obtained by Zhukov et al. [17,18,19,20].

Hereafter, the samples will be referred to by their Co content (in %), followed by *sp* (samples obtained by sputtering) or *ga* (by gas aggregation), e.g., Co3sp is the sample with 3% of Co obtained by sputtering, whereas Co2.5ga is the sample with 2.5% of Co obtained by gas aggregation

The thickness of the sp samples was estimated in 590 nm from the growth rates and the time of growth: However, this approach was not valid for the ga ones, as the combined growth with preformed NPs and sputtered Cu matrix gave place to a very porous and rough film. We have tried to determine its thickness with Atomic Force Microscopy measurements (NT-MDT, Moscow, Russia) but we were not able, given the roughness and irregularity.

X-ray diffraction (XRD) was performed in specular θ–2θ geometry using CuKα radiation in a Brucker D8 Advance system (Bruker, Billerica, MA, USA). Field-cooled (FC) and zero-field-cooled (ZFC) magnetization curves were recorded upon heating from 5 to 375 K using an EverCool MPMS SQUID magnetometer (Quantum Design, San Diego, CA, USA). These ZFC and FC curves were measured after sample cooling in zero field and 100 Oe, respectively. In addition, magnetic hysteresis loops were measured at 300 K up to a maximum applied field of 50 kOe. The diamagnetic contribution corresponding to the Si substrate has been subtracted in all the graphs. The resistance was measured in the temperature range of 5–300 K by a four-probe method in a Quantum Design PPMS System (Quantum Design, San Diego, CA, USA) with the P400 Resistivity Option. This configuration allows taking resistively measurement with accuracy of 10 mkOm that is two order of magnitude lower that the resistance of the samples. The magnetoresistance was measure in transverse configuration.

The magnetoresistance ratio (MR) is defined as:

(1) $$\mathrm{MR}\left(\%\right)=\frac{\Delta R}{R}\left(\%\right)=\frac{R\left(H\right)-R\left(0\right)}{R\left(0\right)}\times 100$$

where R(H) is resistance at given magnetic field, H, whereas R (0) is the resistance at H = 0 Oe.

## 3. Results and Discussion

### 3.1. X-ray Diffraction

**Figure 2:**
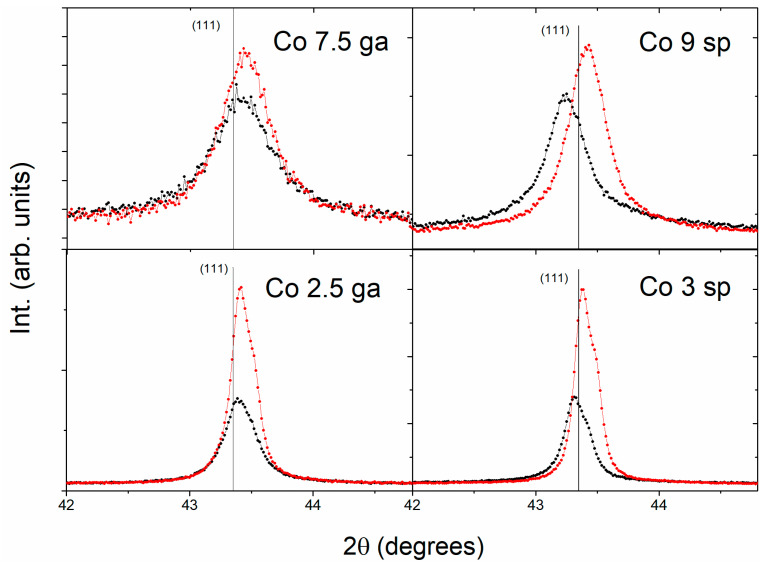
Detail of the X-ray diffractograms of the samples, as-deposited (empty black circles) and after annealing (red solid circles). The vertical lines indicate the position of the reflections of pure Cu fcc.

The X-ray diffractograms of all the samples have been obtained. In all the samples, only reflections of a mixed Cu-Co fcc phase are observed, showing a textured growth in the (111) direction, typical of fcc sputtered films. Hence, a detail corresponding to the first reflection is shown in Figure 2. No reflections of Co (either fcc or hcp) are found, not even after annealing (which usually favors the segregation of the Co from the Cu matrix). This is likely due to the small size of the Co clusters, giving rise to a distortion of the Cu matrix [27] or very small peaks (overlapping with those of the Cu matrix). In the as-deposited samples, the position of the peak is displaced with reference to the position of pure Cu fcc (indicated by a vertical line in the picture). In the case of *ga* samples, the peak is displaced slightly to the right (i.e., corresponding to a smaller lattice parameter) whereas in the sp ones is displaced to the left (greater lattice parameter), which is not what we could expect applying Vegard’s law (given the fact that in the sp samples there is likely a solid solution of Cu–Co, the smaller size of Co compared to Cu would produce a decrease in the lattice parameter). Therefore, that increase in the lattice parameter is due to stress in the thin films, as has been also observed in other granular alloys (e.g., in Co–Cu granular alloys obtained by pulsed laser deposition [28]). We have checked that pure Cu thin films deposited on the same conditions do not present that shift of the peak (figure not shown), pointing out that the stress is induced by the codeposition of Co or the deposition of the preformed Co clusters. Focusing on the annealing, it only gives rise to an increase in crystallinity and almost negligible changes in the position of the reflections in *ga* samples and a more remarkable shift to the right of the peak in sp ones, due to the relaxation of the stress and the segregation of the diluted Co in the matrix, which do not occur in the *ga* samples, where the Co clusters are preformed.

### 3.2. Magnetic Properties

**Figure 3:**
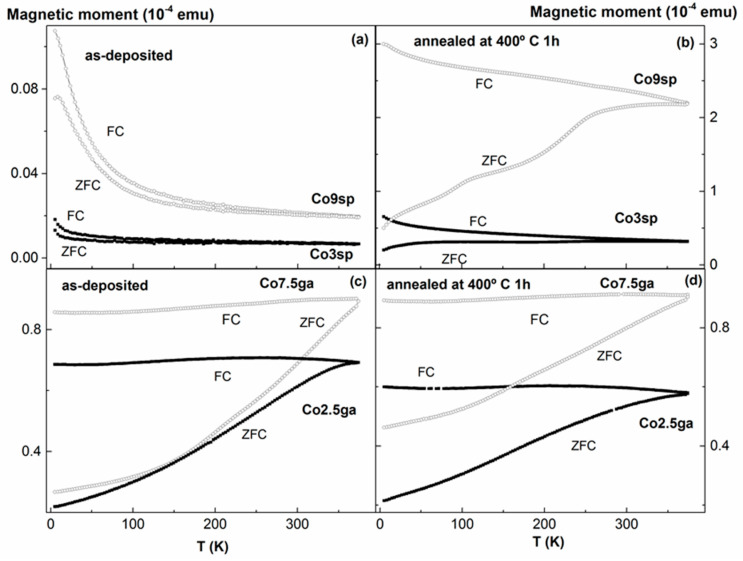
ZFC-FC curves, measured under an applied field of 100 Oe, of the samples obtained by sputtering as-deposited (**a**) and annealed (**b**), and obtained by sputter gas aggregation, as-deposited (**c**) and annealed (**d**).

Figure 3 displays ZFC and FC magnetization curves of all the samples. In all the cases a clear irreversibility between both curves is observed. With regard to the sputtered as-deposited samples (see Figure 3a), a sharp increase of the magnetic moment at very low temperatures is found (in both curves). Such a temperature dependence of the magnetic moment can be attributed to the paramagnetic (PM) or superparamagnetic (SPM) contribution of diluted Co atoms (or very small Co clusters) in the Cu matrix, as it could be expected given the preparation method. In the case of the Co9sp sample, a peak is hinted in the ZFC curve at 8 K. These kind of peaks in the ZFC curves is usually related to the blocking temperature, T_B_, of superparamagnetic (SPM) NPs. In the present case of immiscible Co–Cu alloy, nano-sized Co or Co-rich precipitations are expected. The condition for superparamagnetic behavior of spherical NPs presenting uniaxial anisotropy can be expressed as [29]:

(2) $$K\times V=25k_{B}T_{B}$$

where V is the volume of the nanoparticle, K is the anisotropy energy, and k_B_ is the Boltzmann constant. If we try to use this equation to estimate the size of the Co SPM NPs, the problem would be the adequate determination of the constant anisotropy, which can be fairly different in the case of thin films or NPs from the bulk value, even more so if there is some kind of alloying (as quite likely is our case) [30]. However, it is straightforward to relate the T_B_ with the size of those NPs (the smaller the T_B_, the smaller the size), supposing that there is no interaction (or very small one) between them. Hence, in this case, with such a small T_B_, the Co SPM NPs size should be really small.

After annealing (one hour at 400 °C, 673 K, the shape of the curves changes strongly (see Figure 3b). The most reasonable explanation of such changes is the segregation of the Co from the Cu matrix, giving place to bigger clusters. Hence, the magnetic moment increases strongly, there is no longer a sharp increase at low temperatures, and one or two peaks are hinted in the ZFC curve, suggesting the presence of bigger Co (or Co-rich) clusters.

Given the preparation method of the other samples (combination of preformed Co NPs with a Cu matrix), the as-deposited state of those samples present already quite big Co NPs, so big indeed that they present a FM behavior instead of a SPM one (as commented in the experimental section, the size of the Co NPs in the GA samples is about 11 nm in diameter whereas the critical diameter for spherical Co NPs for SPM behavior at room temperature is about 8 nm [29]). This agrees well with what is observed in Figure 3c: the absence of clear peak in the ZFC curve, associated to a blocking temperature, in the measured temperature range. Even more, the annealing at 400 °C gives place just to negligible changes in the shape and values of the curves (see Figure 3d). This is reasonable, given the fact that the annealing temperature is not too high and that almost all the Co is already forming nanoclusters. Hence, a higher temperature would be required to give place to the aggregation of those NPs.

**Figure 4:**
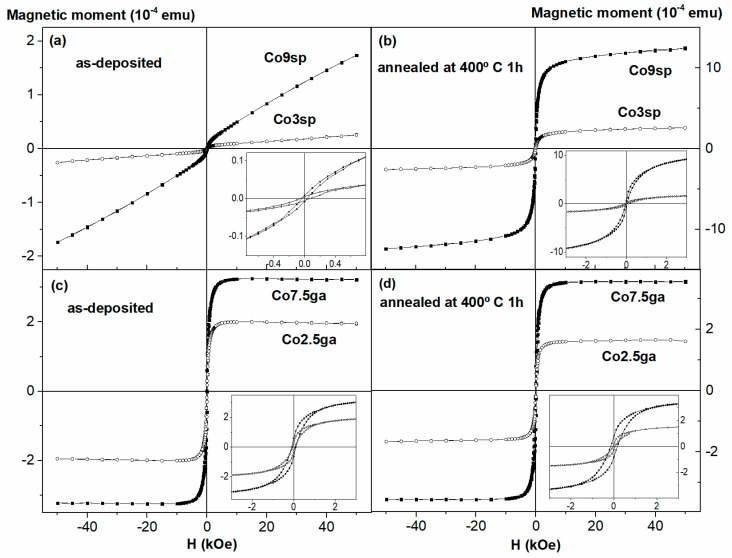
Hysteresis loops at room temperature of the samples obtained by sputtering, as-deposited (**a**) and annealed (**b**), and obtained by sputter gas aggregation, as-deposited (**c**) and annealed (**d**).

The hysteresis loops at room temperature of all the samples are shown in Figure 4. In the case of the sputtered as-deposited samples, mainly a SPM/PM behavior is observed (no saturation even at 50 kOe, very small hysteresis), as can be seen in Figure 4a. After the annealing (Figure 4b), the shape of the loops evolves towards that of a typical ferromagnetic (FM) material, although there is still a certain SPM/PM contribution, pinpointed by the no saturation of the loops. Meanwhile, the samples obtained by gas aggregation, exhibit typical FM behavior, even in the as-deposited state (see Figure 4c). As in the case of the ZFC-FC curves, the annealing of these samples remarkably changes the hysteresis loop shape and the magnetic moment values. All these changes agree well with what we observed in the ZFC-FC curves and with the known influence of the preparation method.

**Figure 5:**
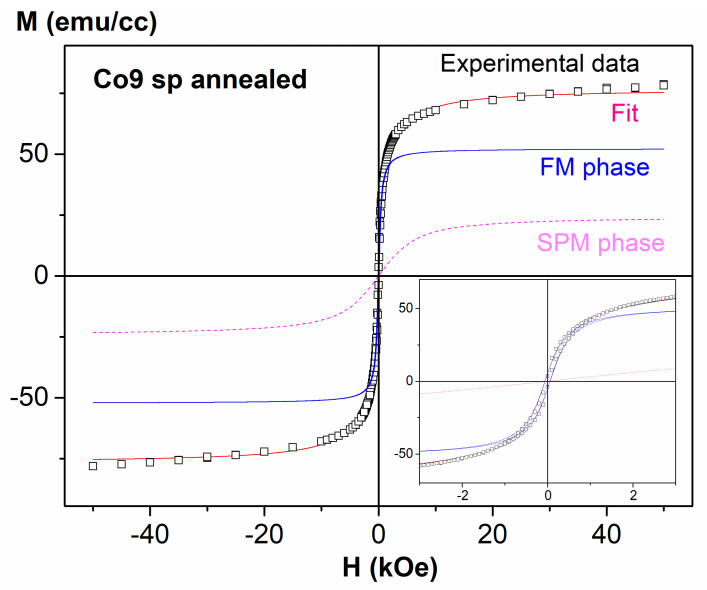
Example of the fit obtained for one of the samples (Co9sp annealed) considering a superparamagnetic (SPM) phase (adjusted by a Langevin function) and a FM phase. Inset: detail at low fields.

In order to obtain additional information from the hysteresis loops, we have analyzed them considering just one FM phase (in the case of the GA samples) and both a FM and a SPM phase in the case of the SP ones. The expression used is the following [31]:

(3) $$M\left(H\right)=\frac{2M_{S}^{\mathrm{FM}}}{\pi }\arctan\left\{\frac{H\pm H_{C}}{H_{C}}\tan\frac{\pi S}{2}\right\}+M_{S}^{\mathrm{SPM}}L\left(\frac{\mu H}{\mathrm{kT}}\right)$$

where the first term is the usual empirical expression to represent a FM hysteresis curve [31] and the second term corresponds to the SPM one. In particular, $M_{S}^{\mathrm{FM}\left(\mathrm{SPM}\right)}$ is the saturation magnetization of the FM (SPM) phase, $H_{C}$ is the coercive field, s is the squareness of the FM loop (i.e., the ratio of the remanent magnetization to $M_{S}^{\mathrm{FM}}$), and $L\left(\frac{\mu H}{\mathrm{kT}}\right)=\cot h\left(\frac{\mu H}{\mathrm{kT}}\right)-{\left(\frac{\mu H}{\mathrm{kT}}\right)}^{-1}$ is the well-known Langevin function, being µ the average moment per SPM NP, k the Boltzmann constant, H the applied magnetic field and T the temperature. Figure 5 shows an example of the obtained fit for one of the samples.

Table 1 summarizes the results obtained from the fits of the hysteresis loops of the sp samples. In particular, the mean diameter D has been obtained considering spherical nanoparticles, according to the expression:

(4) µ=MSCofccV=MSCofcc×πD36

where $M_{S}^{\mathrm{Cofcc}}$ is the saturation magnetization of Co fcc. The percentage of Co in the SPM(FM) phase has been estimated from $M_{S}^{\mathrm{SPM}\left(\mathrm{FM}\right)}/M_{S}^{\mathrm{Cofcc}}$ (normalized by the % of Co), i.e., we have considered that if all the Co in the sample contributed magnetically, the saturation magnetization of the sample would be that of bulk fcc Co (normalized to the amount of Co in the sample). The remaining percentage represents the amount of Co atoms not giving place to a magnetic contribution because they are too diluted in the Cu matrix.

As can be observed, both sputtered as-deposited samples exhibit very small values of D and µ (below 1 nm and 200 µ_B_, respectively). These values are mean values, so probably there is a certain distribution, but, anyway, the SPM NPs in both samples are fairly small, agreeing well with the sharp increase at low temperatures in the ZFC curves (with a peak hinted at about 8 K in the Co9sp sample). Additionally, the % Co in the FM phase in both as-deposited samples is almost negligible (as could be expected). In fact, probably that FM contribution is not totally related to big FM NPs, but is related to dipolar interactions between Co SPM NPs [32]. It is also noteworthy that in both cases, most of the Co atoms (about 70–80%) are diluted and not contributing to the magnetic properties of the samples.

As we anneal, there is a segregation of part of this diluted Co atoms, giving rise to greater values of D (about 2 nm) and to a marked increase of the % of the FM phase (up to 14.6 for the Co3sp ann sample and 40.1 for the Co9sp one). The variation of the amount of Co contributing to the SPM decreases very slightly and the amount of diluted Co (not magnetically contributing) decreases strongly.

The obtained values of the percentage of Co in the different phases (SPM, FM and diluted) and their evolution after the annealing agree well with the values obtained by other authors in Co–Cu granular alloys fabricated by other techniques (electrodeposition, laser ablation, melt-quenching) [7,8,32], although, evidently, there are some differences given the different fabrication techniques (hence, in our case, the % in diluted phase is higher than in the electrodeposited or melt-quenched samples but lower than in the samples obtained by laser ablation). However, the value of D for our as-deposited samples is smaller than in the samples obtained by other techniques. On the other hand, Ustinov et al. [23] grew Fe/Cr multilayers with very thin Fe layers (so thin indeed that in some cases it was quite similar to a granular alloy) and their estimated values of D were about 0.5–0.7 nm in the case of the thinnest Fe layers, which are more similar to our values. Lobov et al. [22] grew Co/Cu superlattices with very thin Co layers, in a similar way to the work of Ustinov but with Co/Cu instead of Fe/Cr, and they estimated µ to be 600 µ_B_ for the sample with Co layers of 0.3 nm of thickness, a sample with a non-negligible FM contribution, and said that their samples with thinner Co layers have a practically SPM behavior (although they did not fit them, that would imply quite likely smaller values of µ), which agrees quite well with our results.

Meanwhile, the loops of the ga samples are fitted successfully with just a FM contribution (as could be expected, just seeing the shape of the loops). Therefore, in this case there is no Co atoms behaving in a SPM way nor diluted atoms not magnetically contributing (or, if there are any, practically in a negligible amount).

### 3.3. Magnetotransport Properties

**Figure 6:**
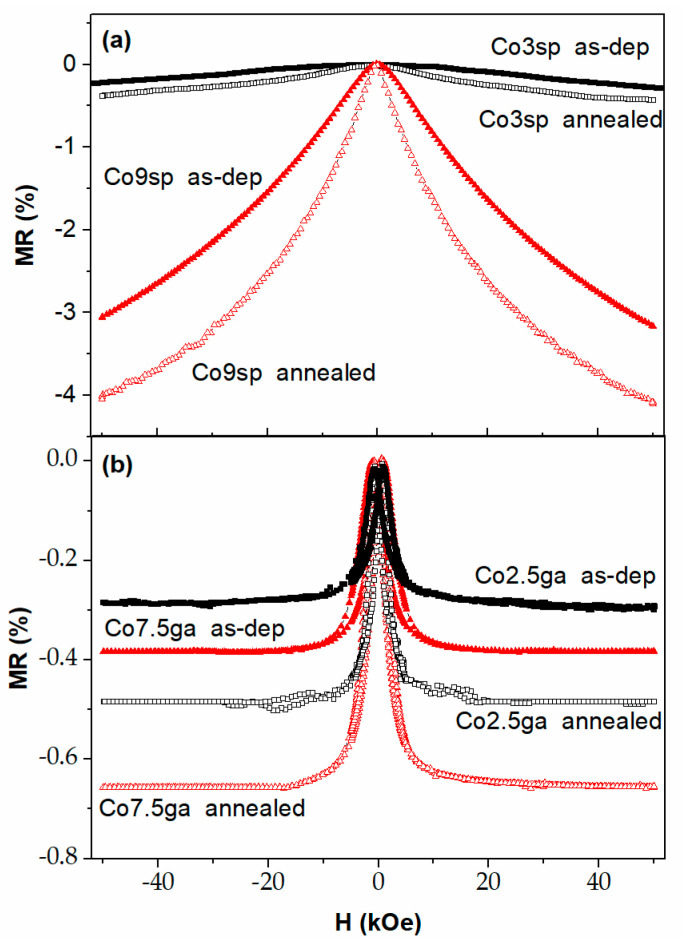
Magnetoresistance loops of the samples obtained by sputtering (**a**) and sputter gas aggregation (**b**), in both cases as-deposited and annealed.

Figure 6 shows the magnetoresistance loops at 10 K of all the samples, with an applied field up to 50 kOe. For all the as-deposited samples with low Co content (2.5% or 3%), the MR are quite low (below 0.4% in absolute value). The main difference between the samples obtained by the sputtering and gas aggregation is that in the latter, MR saturates at about 10 kOe, whereas in the former keeps increasing up to 50 kOe. Meanwhile, after annealing, there is an increase of the MR, but keeping the same shape and with relatively low MR values (below 0.5% in absolute value). Meanwhile, in the case of the samples with “high” Co content (7.5 or 9%), in addition of the difference in the shape already commented, the values before annealing are also quite different: 0.4% for the co7.5ga sample, 3% for the co9sp one (both in absolute value). After the annealing, the behavior is the same as observed in the other samples: the same shape, with an increase in the absolute value of MR values (0.65%—co7.5ga; 4%—co9sp). Hence, we can follow indirectly the relevance of the preparation method in the microstructure, and, therefore, in the magnetic and magnetotransport properties. In the sputtered samples, initially we have a solid solution of Co–Cu, with many Co atoms (and small clusters) diluted in the Cu matrix. As we increase the Co content, there is a higher density of Co clusters (and some of them are bigger), giving place to a higher (in absolute value) MR, as GMR in heterogeneous alloys is affected, between other factors, by the size and density of the FM NPs) [33]. In the case of gas-aggregated samples, the Co NPs have been preformed and have relatively big sizes. Therefore, the density is going to be lower, which can partially explain the low MR values obtained. Additionally, the annealing is not enough to agglomerate the NPs and do not affect too much the GMR. In fact, there is a small increase, perhaps due to a change in the spin-dependent scattering roughness of the interface Co/Cu [33].

**Figure 7:**
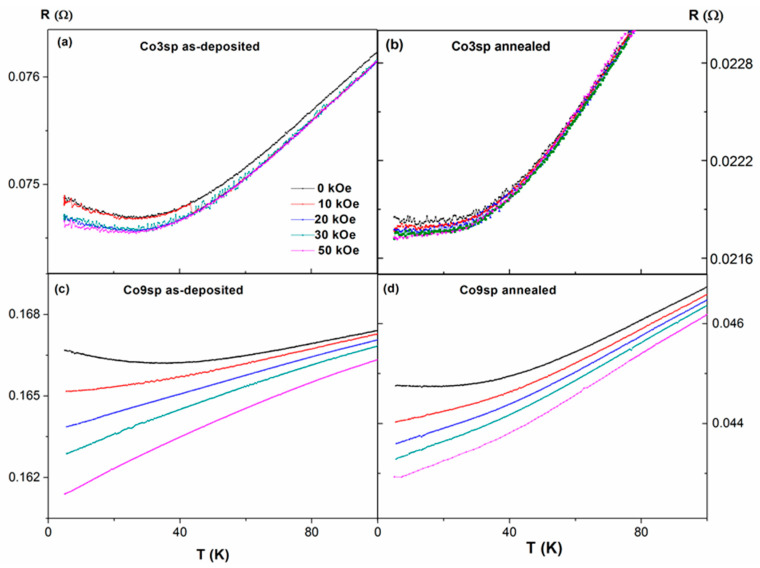
Effect of magnetic field on temperature dependence of resistance, R, in all the samples obtained by sputtering, as-deposited (**a**,**c**) and annealed (**b**,**d**).

**Figure 8:**
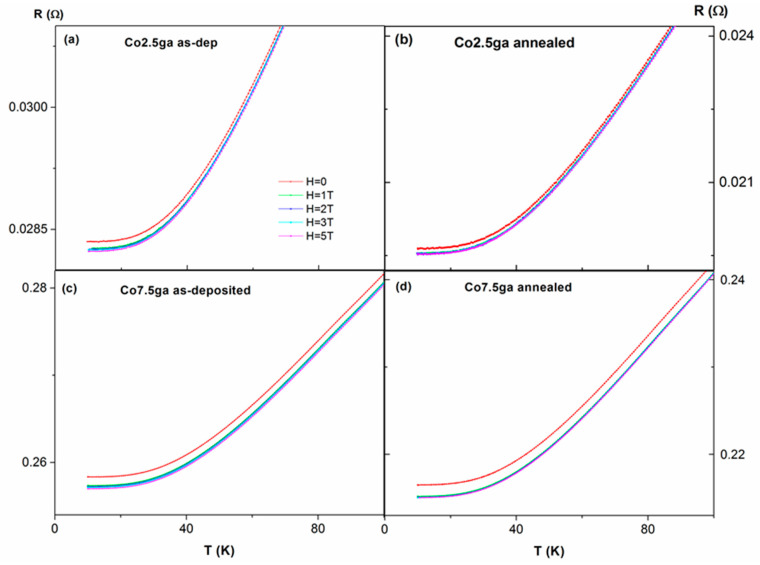
Effect of magnetic field on temperature dependence of resistance, R, in all the samples obtained by gas aggregation, as-deposited (**a**,**c**) and annealed (**b**,**d**).

In order to check the existence of a resistance minimum (linked to a resistivity minimum) versus the temperature, R(T) dependences were measured under different applied fields, H, (see Figure 7 and Figure 8). First, we focus on the samples obtained by sputtering (Figure 7). In both as-deposited samples (Co3sp and Co9sp), a clear minimum in R(T) is found, at 28 K (sample Co3sp) and 36 K (Co9sp), at H = 0. After annealing, the minimum temperature decreases (to 13 and 17 K respectively) and is not so clearly marked (i.e., the slope of the resistance below the minimum is less pronounced). Usually, such a minimum in R(T) is attributed to the Kondo effect related to the effect of magnetic impurities on electron scattering [18]. However, in classical Kondo-systems, the required content of magnetic impurities is fairly small (0.002–0.02%), so that the impurities are well separated and are able to react independently to the spin of the conduction electron [21]. This is far from being the case in our samples, with Co content in the range of 2.5–9%. Nevertheless, the preparation method can strongly affect the distribution of the Co within the Cu matrix. In fact, in the samples obtained by sputtering, we expect that there are Co-rich regions (or even small Co clusters, even in the as-deposited case) but also Co-poor regions, with just very small clusters or just Co atoms diluted in the Cu matrix (in fact, from the fit of the hysteresis loops, we deduce that a high percentage of Co atoms are very diluted in the Cu matrix, agreeing well with these Co-poor regions). These Co-poor regions would be fairly more similar to a classical Kondo system. These results are relatively similar to the results of Lobov et al. in Co/Cu superlattices with a ultrathin Co layer of 0.03 nm of thickness (although in that case there was still a clear minimum with applied fields) [22]. However, this Kondo-like behavior is more marked in Fe/Cr multilayers with ultrathin Fe layers [23] or in the Co–Cu granular alloys obtained by melt-quenching [16,17,18,19,20]. Additionally, considerable effect of annealing on R(T) dependence is consistent with changes in magnetic moment versus temperature dependencies and modifications of the hysteresis loops: all the experimental results must be attributed to either Co segregation or precipitations of Co from Co–Cu solid solution. As we have deduced from the fit of the hysteresis loops, there is a clear decrease in the amount of Co atoms diluted in the Cu matrix and not magnetically contributing (the most likely candidates for giving rise to this Kondo-like effect), so the decrease of the intensity of that effect seems quite reasonable. On the contrary, the samples obtained by gas aggregation, with preformed Co NPs are quite far from a classical Kondo system, explaining the absence of a minimum in the resistance.

Given the fact that the vanishing of the R(T) minimum under the application of an external magnetic is one of the typical features of the classical Kondo effect [19,34], we have studied the influence of the magnetic field on the R(T) dependence for all the samples, applying magnetic fields in the range from 10 to 50 kOe (see Figure 7). We find two different behaviors in our samples: the minimum observed in the sample Co3sp is negligibly affected by the magnetic field (before and after annealing), whereas in the case of the sample Co9sp, the minimum disappears as we apply the magnetic field (even under the lower applied field of 10 kOe).

Meanwhile, in the case of the samples obtained by gas aggregation (see Figure 8), there is no clear minimum in any of the samples, both as-deposited and annealed. In fact, what we find is a plateau at lower fields, not affected in its shape by the applied field (a similar behavior to the case of a thin film of pure Cu -figure not shown-).

**Figure 9:**
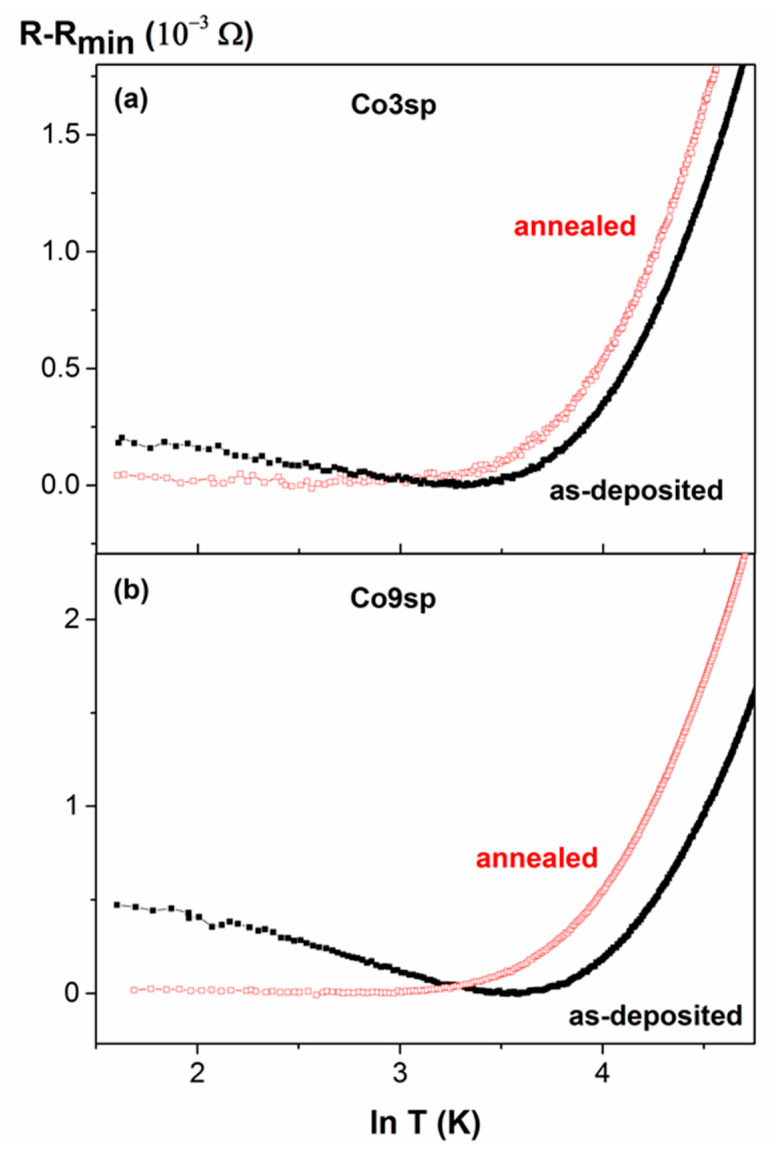
R-Rmin(ln T) dependence for the samples obtained by sputtering, as-deposited (black solid squares) and annealed (red empty squares). (**a**) Co3sp sample; (**b**) Co9sp sample.

Another typical feature of the classical Kondo effect is as resistivity contribution behaving as ln(T) [17,20]. Therefore, we have plotted R-R_min_ versus ln (T) for the samples obtained by sputtering (as-deposited and annealed) and shown that in Figure 9. As can be seen, the sample Co3sp follows a quite linear behavior with ln (T) (both for as-deposited and annealed state), whereas the sample Co9sp does not follow so well the linear behavior (especially in the as-deposited case). In both annealed cases, the slope is fairly small.

As we have seen, the samples obtained by gas aggregation do not present a clear minimum in the resistance whereas those obtained by sputtering present one, showing several features typical of a classical Kondo system, as the vanishing of the minimum as we apply a high magnetic field or the approximate low temperature ln T dependence of the resistance. However, this agreement is only partial: in the case of the Co3sp sample, the effect of the magnetic field on the minimum is practically negligible, but, on the other hand, the resistance follows quite well a logarithmic dependence with temperature at low temperatures. Meanwhile, in the case of the Co9sp sample, the effect of the magnetic field is the expected one in Kondo effect but the resistance does not follow very well the expected logarithmic dependence with temperature. In addition of classical Kondo effect, there are other mechanisms that could give rise to such a minimum, as weak location, enhanced electron-electron interaction, two-channel Kondo (i.e., scattering of electrons by structural two-level systems, TLS), and scattering of strongly spin-polarized charge carriers on diluted magnetic moments [35,36,37]. The fact that the minimum is affected by the magnetic field (at least for the sample Co9sp) supports the classical Kondo contribution [20] but the Kondo temperature of Co in Cu is of the order of 1000 K, according to theoretical calculations [38,39], which would rule out this possibility. Saito et al. [35,40] explained such a minimum in Co/Cu multilayers as a combination of the T dependence of saturation resistivity and that of the GMR. However, in that case, the multilayer geometry was key for explaining the behavior of the GMR (with weak interlayer exchange coupling), which is not our case.

On the other hand, the non-homogeneous Co distribution and atomic disorder could give rise to some of the other mechanisms, but, alas, it is not yet crystal-clear which mechanism is giving rise to this minimum. However, all the experimental results and especially the remarkable effect of preparation method and annealing on the magnetic and transport properties point out on importance of Co distribution in Co–Cu matrix.

## 4. Conclusions

We have studied the structural, magnetic, and magnetotransport properties of Cu_100−x_Co_x_ thin films obtained by sputtering (x = 3, 9) and sputter gas aggregation (x = 2.5, 7.5), taking into account the effect of annealing (1 hour at 400 °C). The role of the fabrication method in the properties is key. Hence, the as-deposited samples contain small Co (or Co-rich) clusters, but these are greater in the samples obtained by gas aggregation (with ferromagnetic behavior at room temperature) whereas in the samples obtained by sputtering, the size of the clusters decrease, also with diluted Co atoms in the matrix. The annealing affects negligibly the samples obtained by gas aggregation, but the ones obtained by sputtering are more affected, with greater clusters as the Co segregates from the matrix. This behavior is also reflected in the magnetoresistance (MR) measurements of the samples: a more lineal behavior for the samples obtained by sputtering, saturation at relatively low fields (about 10 kOe) for the other samples. Finally, a Kondo-like minimum in the resistance versus temperature is only found in the samples obtained by sputtering, affected both by the magnetic field and the annealing. The observed Kondo-like behavior is explained by the presence of diluted Co atoms in the Cu matrix of sputtered samples. The effect of annealing on the Kondo-like minimum is attributed to the precipitation of diluted Co atoms from Co–Cu solid solution.

## Figures and Tables

**Table 1:** Mean magnetic moment, µ, and diameter, D, and percentage of Co atom in the SPM (FM) phases for the as-deposited and annealed sp samples.

Sample	µ (µ_B_)	D (nm)	% SPM	% FM
Co3 sp a-d	30	0.7	28.2	0.9
Co 3 sp ann	680	2.0	27.7	14.6
Co9 sp a-d	120	0.9	19.3	0.7
Co9 sp ann	1700	2.2	18.8	40.1

## Data Availability

The data presented in this study are available on request from the corresponding authors.
